# Using phenotypic data from the Electronic Health Record (EHR) to predict discharge

**DOI:** 10.1186/s12877-023-04147-y

**Published:** 2023-07-11

**Authors:** Monisha C. Bhatia, Jonathan P. Wanderer, Gen Li, Jesse M. Ehrenfeld, Eduard E. Vasilevskis

**Affiliations:** 1 Vanderbilt University School of Medicine, 1161 21St Ave S, Nashville, TN 37232 US; 2 Current Address: University of California San Francisco, 500 Parnassus Avenue, San Francisco, CA 94143 US; 3 Department of Anesthesiology, Vanderbilt University Medical Center, 1211 Medical Center Drive, Nashville, TN 37232 US; 4 Department of Biomedical Informatics, Vanderbilt University Medical Center, 1211 Medical Center Drive, Nashville, TN 37232 US; 5 Department of Surgery, Vanderbilt University School of Medicine, 1211 Medical Center Drive, Nashville, TN 37232 US; 6 Department of Health Policy, Vanderbilt University School of Medicine, 1211 Medical Center Drive, Nashville, TN 37232 US; 7 Current Address: Medical College of Wisconsin, 8701 Watertown Plank Rd, Wauwatosa, WI 53226 US; 8 Department of Medicine, Section of Hospital Medicine, Division of General Internal Medicine and Public Health, , Vanderbilt University Medical Center, 1211 Medical Center Drive, Nashville, TN 37232 US; 9 Geriatric Research, Education and Clinical Center (GRECC), VA Tennessee Valley Healthcare System, 1310 24Th Ave S, Nashville, TN 37212 US; 10 Center for Quality Aging, Department of Medicine, Vanderbilt University Medical Center, 1211 Medical Center Drive, Nashville, TN 37232 US; 11 Center for Clinical Quality and Implementation Research, Vanderbilt University Medical Center, 1211 Medical Center Drive, Nashville, TN 37232 US

**Keywords:** Post-acute care, Prediction models, Frailty, Functional status, Health systems

## Abstract

**Background:**

Timely discharge to post-acute care (PAC) settings, such as skilled nursing facilities, requires early identification of eligible patients. We sought to develop and internally validate a model which predicts a patient’s likelihood of requiring PAC based on information obtained in the first 24 h of hospitalization.

**Methods:**

This was a retrospective observational cohort study. We collected clinical data and commonly used nursing assessments from the electronic health record (EHR) for all adult inpatient admissions at our academic tertiary care center from September 1, 2017 to August 1, 2018. We performed a multivariable logistic regression to develop the model from the derivation cohort of the available records. We then evaluated the capability of the model to predict discharge destination on an internal validation cohort.

**Results:**

Age (adjusted odds ratio [AOR], 1.04 [per year]; 95% Confidence Interval [CI], 1.03 to 1.04), admission to the intensive care unit (AOR, 1.51; 95% CI, 1.27 to 1.79), admission from the emergency department (AOR, 1.53; 95% CI, 1.31 to 1.78), more home medication prescriptions (AOR, 1.06 [per medication count increase]; 95% CI 1.05 to 1.07), and higher Morse fall risk scores at admission (AOR, 1.03 [per unit increase]; 95% CI 1.02 to 1.03) were independently associated with higher likelihood of being discharged to PAC facility. The c-statistic of the model derived from the primary analysis was 0.875, and the model predicted the correct discharge destination in 81.2% of the validation cases.

**Conclusions:**

A model that utilizes baseline clinical factors and risk assessments has excellent model performance in predicting discharge to a PAC facility.

**Supplementary Information:**

The online version contains supplementary material available at 10.1186/s12877-023-04147-y.

## Background

Timely discharge planning (DP) is critical to successful transitions of care from the hospital to the post-acute care (PAC) setting. PAC settings include Skilled Nursing Facilities, Long Term Acute Care facilities, and Inpatient Rehabilitation. Previous studies suggest that early DP decreases hospital length of stay and readmissions [1]. Decision-making regarding discharge destination, however, may occur late during hospitalization, leaving little time to improve the transition to PAC [2, 3].

Availability of a tool to predict discharge destination early in the hospitalization may improve transitions of care to PAC facilities by enabling social services to contact facilities, coordinate insurance authorizations, engage physical therapy to conduct a timely assessments, and aid the primary team in tailoring the patient’s discharge planning for PAC [4, 5] Currently, there are a small number of predictive models, with only one of these using data from the first day of admission [6–8]. Other models focus on a.narrow range of clinical specialties including cardiac surgery, [9] orthopedics, [10, 11] and trauma [12]. However, there are few models which could be applied across an entire hospital population.

An ideal prediction tool for broad clinical application would automatically generate a score soon after admission, using objective and routinely available data from the electronic health record (EHR). Improvements in bioinformatics now allow for automated analysis of data routinely collected and collated in the EHR. Such EHR-derived scores have been developed for prediction of readmission [13] and physiologic deterioration [14]. The primary aim of this study was to develop and validate an EHR-derived model that produces a score predictive of discharge to PAC, using data readily available within 24 h of admission.

## Methods

### Study design

After obtaining approval from the Institutional Review Board at our institution, we conducted a retrospective cohort study with a validation data set. The Transparent Reporting of a Multivariable Prediction Model for Individual Prognosis or Diagnosis (TRIPOD) guidelines were used in the planning and execution of the study [15].

### Study setting and participants

We included all adults (≥ 18 years of age) who were admitted for an inpatient stay at Vanderbilt University Medical Center from September 1, 2017, to August 1, 2018. An individual patient could account for multiple admissions during this time period. We excluded patients admitted for “observation”, patients transferred to another acute care hospital over the course of their admission, patients discharged on the day of admission, and patients who left the hospital against medical advice. Patients who were transferred by a court or law enforcement or could not have their admission source or discharge destination identified were excluded (Additional File 1).

### Data source

We extracted study data from the hospital data warehouse (HDW), a de-identified database of adult hospital patient information, using structured query language (SQL). HDW information mirrors clinical data from the EHR system, Epic©, (Epic Systems Corporation, Verona, WI), and includes data from all patients, not just those who are admitted for operative management.

### Model candidate predictors

Predictor variables were generated and screened based on a literature review of risk factors predictive of discharge destination which are also collected routinely by the EHR early in the course of hospitalization (< 24 h from admission time). Based on a literature review we identified candidate variables including age, [7, 8, 10, 16] gender, [9] insurance status, pre-hospital location [7], admission source, admission service, and markers of frailty [6–8, 10] (Table 1).

**Table 1:** Candidate variables assessed for inclusion in our post-acute care prediction model

**Demographics**	Age Gender Race Health Insurance Pre-Hospital Location
**Markers of Comorbidities**	Body mass index Cognitive decline index (MMSE/RASS) Number of hospitalizations in previous 2 years Number of Medications Admission Unit (Surgical vs. Medical vs. ICU)
**Nursing Data**	Braden Score Fall Risk Assessment Score
**Markers of Illness Severity**	Vital Signs Arterial/Venous Blood Gas Electrolytes Liver Function Tests Complete Blood Count Coagulation Studies Glasgow Coma Scale Score Number of medications on the Pre-Hospital List
**Covariant Data**	Health Insurance Information Pre-hospital Location Health Insurance

The primary type of health insurance plan [7, 10, 16] for each patient was categorized as, 1) Medicare (which included patients whose primary insurance type was Medicare or Medicare Advantage); 2) Medicaid (which included patients whose primary insurance type was Medicaid and TennCare); 3) Private; and 4) Self-pay/Other (which included patients who paid the bill on their own or their insurance information was unknown). For patients covered by multiple insurance plans, we used the first presented insurance during the admissions. The patient’s pre-hospital location [6] was divided into three categories: Home, Outside Facility (which included SNFs, Long Term Acute Care facilities, and Inpatient Rehabilitation), or Physician/Clinic Office. The admission source was grouped into two categories: Admitted through the Emergency Department versus other (e.g., direct admission, transfer). Admission services were categorized into intensive care unit (ICU), obstetrics/gynecology, and medical/surgical. Six services qualified as ICU: Trauma, Burn, Cardiac, Neurological, Medical, and Surgical Intensive Care Units. Labor and Delivery, Post-partum, Maternal Care, and Women’s Surgery were all considered obstetrics/gynecology admissions. The remaining admissions were considered general medical/surgical.

Factors that reflected the presence of geriatric syndromes included the Braden Score [17], Morse Fall Risk Score [18], and polypharmacy [12, 19]. The Braden Score, ranging from less than or equal to 9 to as high as 23, is a nursing assessment performed after admission to determine a patient’s risk of developing pressure ulcers [20]. Braden score has been shown to be associated with discharge location [21, 22]. We retrieved measurements from the first evaluation after admission, and the maximum and minimum values within 24 h when multiple measurements were available. Similar to Braden Score, Morse Fall Risk Score is another nurse-reported patient assessment, with a range of 0 to 125. The first, minimum and maximum fall risk measurements within 24 h after admission were obtained. Both Braden Score and Morse Fall Risk Score were treated as continuous variables, and simple imputations of median values were imputed for missing data. Pre-hospital medications was defined as a count of all medications the patient was taking before hospital admission, as entered by the primary treatment team or pharmacist as part of the admission medication reconciliation. These included medications taken as needed, on a short-term basis, and topically.

### Primary outcome

Discharge destination was classified into two categories: PAC (rehabilitation facility, skilled nursing facility, long term acute care) versus all other discharges that may include home, hospice, and deceased. We included hospice and deceased patients in the cohort but not the outcome definition to improve the model’s real-world applicability for identifying PAC discharges. The primary event of interest of this study was discharge to PAC versus non-PAC discharge.

### Statistical analysis

Demographic and clinical variables were used to characterize the study sample with means and standard deviations (SDs) for parametric variables, with medians and interquartile ranges (IQRs) for nonparametric variables and with percentages for categorical variables, as appropriate.

The entire cohort was randomly split into a derivation and a holdout group. The derivation cohort was used to examine the association of each potential factor with discharge destination, and the holdout cohort was used to validate the model’s performance. Given the imbalanced ratio of discharges to PAC relative to discharges to home, a random undersampling approach was applied to the derivation cohort for developing the best fit model without introducing bias into the covariates’ parameter estimates.^25,26^ The parameter estimates, odds ratios, and their confidence intervals of covariates are unaffected by the stratified sampling methods, while the intercept parameter estimate is the only part in the model that is affected by the resampling.

Based on plausibility, pragmatism, and availability within 24 h of admission, we first conducted univariate screening for candidate predictors (Table 1) using an uncorrected chi-square test for categorical variables or a Mann–Whitney test for ordinal and continuous variables. A restricted cubic splines approach was applied for modeling non-linear associations. The primary analysis was performed using multivariable logistic regression. A stepwise selection approach was then applied to identify statistically significant covariates for inclusion in regression model. In order to minimize the risk of overfitting, we limited the number of predictors included in the final model following the rule of no less than 20 subjects per variable [23]. The associations were summarized using the odds ratios (ORs) with 95% confidence intervals (CIs) and tested using the Wald multiple degree of freedom Chi-squared test. The variance inflation factors (VIFs) were computed to detect potential collinearity, by assessing the variance change of an estimated regression coefficient.^27^ A calibration plot was generated to assess goodness of fit.

A secondary analysis was then conducted to evaluate the predictive ability of the model. The validation was performed by applying the model to the randomly selected holdout dataset. We derived a predictive score for each patient using the regression coefficients generated from primary analysis, and a matrix was developed to compare the observed with predicted discharge disposition. Sensitivity, specificity, positive predictive value (PPV), and negative predictive value (NPV) were calculated to characterize the performance of the predictive model we generated in primary analysis. The area under the curve (AUC) of the Receiver Operating Characteristic (ROC) curve was created to assess the discrimination ability of the model A two-sided hypothesis testing with a p-value of less than 0.05 deemed to indicate statistical significance. All statistical programming was implemented in SAS 9.4 (SAS Institute Inc., Cary, NC, USA).

## Results

### Characteristics of study population

Between September 2017 and August 2018, 78,659 visits were retrieved electronically from the HDW. After applying exclusion criteria, 23,566 cases met the inclusion criteria (Additional File 1). Of all eligible cases, 19,363 (82.2%) were discharged home, 3041 (12.9%) were discharged to PAC, 762 (3.2%) died and 400 (1.7%) were discharged to hospice.

In primary analysis, a holdout cohort of 2,000 discharges was randomly selected. The random undersampling approach was then applied to the derivation group and a total of 6,000 cases were selected with the ratio of discharge to Home vs. PAC was 1.22. (Table 2) shows the demographics and characteristics of all three cohorts. In brief, the average age of the entire cohort was 53.6 years (SD = 18.8), study cohort 58.0 (18.9), and the holdout cohort 53.0 (18.9). White patients comprised 77.3% of the entire cohort, 78.2% of study cohort, and 77.2% of holdout group. Approximately 52.8% of the eligible patient encounters were admissions from the Emergency Department. Most patients resided at home immediately prior to admission (67.9%), and most were admitted by Medical/Surgical services (69.9%). In the overall group, 12.9% of patients were discharged to PAC, while in the oversampled derivation sample 45% of patients were discharged to PAC.

**Table 2:** Patient demographics and clinical characteristics of the study sample.

**Variables**	**EntireCohort**	**Study Cohort**	**Holdout Cohort**
	**(*n* = 23,566)**	**(*n* = 6,000)**	**(*n* = 2,000)**
**Age** in years, mean (SD)	53.6 (18.8)	58.0 (18.9)	53.0 (18.9)
**Gender** (%)			
Female	12,309 (52.2%)	2,946 (49.1%)	1,040 (52.0%)
**Race **(%)			
White	18,225 (77.3%)	4,690 (78.2%)	1,543 (77.2%)
African American	3,689 (15.7%)	967 (16.1%)	316 (15.8%)
Others/Unknown	1,652 (7.0%)	343 (5.7%)	141 (7.0%)
**Emergency Admission **(%)			
Yes	12,436 (52.8%)	3,594 (59.9%)	1,036 (51.8%)
**Surgical Case** (%)			
Yes	11,598 (49.2%)	2,963 (49.4%)	1,010 (50.5%)
**Pre-hospital Medication Count**, median (IQR)	13 (7-20)	16 (10-22)	13 (7-19)
**Hospital Length of Stay** in days, median (IQR)	3.7 (2.2-6.5)	5 (2.9-8.9)	3.7 (2.3-6.2)
**First Braden Score**, median (IQR)	20 (18-22)	20 (16-21)	20 (19-22)
**Maximum Braden Score**, median (IQR)	21 (19-22)	20 (17-22)	21 (19-23)
**Minimum Braden Score**, median (IQR)	19 (17-20)	18 (15-20)	19 (17-21)
**First Morse Fall Risk Score**, median (IQR)	35 (20-50)	45 (30-60)	35 (20-50)
**Maximum Morse Fall Risk Score**, median (IQR)	45 (35-60)	45 (35-70)	45 (30-60)
**Minimum Morse Fall Risk Score**, median (IQR)	35 (20-45)	35 (20-50)	35 (20-45)
**Admission Source** (%)			
Intensive Care Unit	4,233 (18.0%)	1,412 (23.5%)	332 (16.6%)
Obstetrics and Gynecology	2,841 (12.1%)	490 (8.2%)	250 (12.5%)
Medical/Surgical	16,492 (69.9%)	4,098 (68.3%)	1,418 (70.9%)
**Pre-hospital Location** (%)			
Home	15,993 (67.9%)	3,961 (66.0%)	1,359 (68.0%)
Outside Hospital or Facility	5,149 (21.9%)	1,559 (26.0%)	436 (21.8%)
Physician or Clinic Office	2,424 (10.3%)	480 (8.0%)	205 (10.2%)
**Type of Insurance** (%)			
Medicare	9,552 (40.5%)	3,001 (50.0%)	796 (39.8%)
Medicaid/TennCare	2,800 (11.9%)	606 (10.1%)	223 (11.2%)
Private	7,250 (30.8%)	1,475 (24.6%)	619 (31.0%)
Self-pay/Others	3,964 (16.8%)	918 (15.3%)	362 (18.1%)
**Discharge Destination** (%)			
Home	19,363 (82.2%)	3,300 (55.0%)	1,735 (86.8%)
Post-acute Care	3,041 (12.9%)	2,700 (45.0%)	265 (13.2%)
Hospice	400 (1.7%)		
Deceased	762 (3.2%)		

*SD* Standard Deviation, *IQR* Interquartile Range

### ***PAC-Predict Model Development and Internal Validation (***Fig. 1***)***

**Fig. 1:**
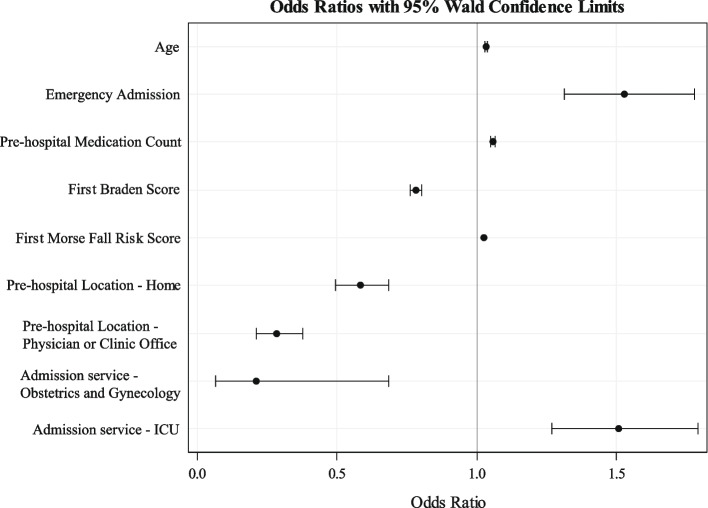
Visualization of the primary analysis results that derived from multivariable logistic regression model. The odds ratio estimates and their corresponding 95% Wald confidence intervals demonstrate the odds of post-acute care discharge associated with the change in the corresponding covariates

**Fig. 2:**
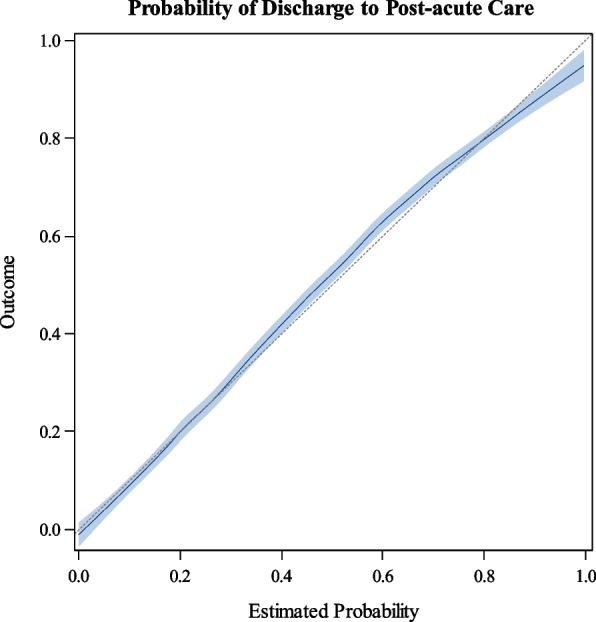
Calibration plot of the model’s predicted probability of PAC discharge. The estimate and 95% upper and lower confidence bounds are represented by the blue line and boundaries, respectively

From the result of primary analysis, we found that older age (adjusted odds ratio [AOR], 1.04 [per year]; 95% CI, 1.03 to 1.04) predicted PAC discharge. Patients admitted through the Emergency Department (AOR 1.53, 95% CI 1.31 to 1.78) had higher odds of PAC discharge. Patients taking more medications (AOR, 1.06 [per one medication increase]; 95% CI 1.05 to 1.07), and higher Morse fall risk scores at admission (AOR, 1.03 [per unit increase]; 95% CI 1.02 to 1.03) were more likely to be discharged to PAC. A histogram of the distribution of medication count is included in Additional File 2A higher Braden score at admission (AOR, 0.78 [per unit increase]; 95% CI 0.76 to 0.80) resulted in lower odds of PAC discharge. Patient admitted from home or the clinic had lower (home—AOR, 0.58; 95% CI, 0.50 to 0.6; clinic—AOR, 0.28; 95% CI, 0.21 to 0.38)) odds of PAC discharge compared to those transferred in from a facility (e.g., hospital). Finally, patients cared for on obstetrics/gynecology teams had reduced odds (AOR, 0.21; 95% CI 0.07 to 0.69), whereas those admitted to ICU teams had elevated odds of PAC discharge (AOR, 1.51; 95% CI, 1.27 to 1.79). The goodness of fit was assessed by calibration plot, which demonstrates that the probability of discharge to PAC was well correlated to the obtained score from the prediction tool (Fig. 2). The AUC was 0.875. For full specifications of the prediction model, see Additional File 3.

**Fig. 3:**
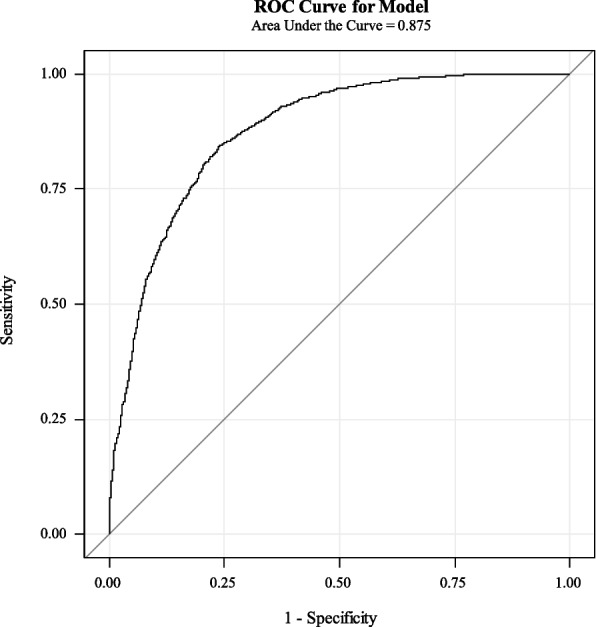
The receiver operator characteristic (ROC) plot for the prediction of discharge to the post-acute care setting

Additional analyses revealed the overall prediction accuracy was 80.3% for the subgroup of discharge to home, 82.3% for the subgroup of discharge to PAC facility, and 80.6% for the entire group. Meanwhile, with the Youden index of 0.63 for optimal cut-point, the sensitivity, specificity, PPV, and NPV of the derived modeling on internal validation cohort were 82.3%, 80.3%, 38.9%, and 96.7%, respectively (Table 3). The ROC plot shows that the area under the curve was 0.875, where a value of 1 would represent a perfect predictive tool, highlighting excellent model discrimination ability (Fig. 3).

**Table 3:** Performance matrix of implementing the predictive model on validation cohort

Predicted Discharge Disposition	Observed Discharge Disposition		
Frequency (N)	Post-acute Care	Home	
Post-acute Care	218	342	Positive Predictive Value = 38.9%
Home	47	1393	Negative Predictive Value = 96.7%
Total	Sensitivity = 82.3%	Specificity = 80.3%

We conducted a post-hoc sensitivity analysis to assess the predictive performance of our model on medical versus surgical patients (Additional File 4), and obstetric/gynecology patients versus those who were not (Additional File 5). Performance between medical and surgical patients was similar. Discharge to PAC for obstetric/gynecology patients is a rare event, hence the model could not assess positive predictive value in the validation cohort. We also assessed how the model performed among younger (< 55 and 56–64 years old) and older (65–79 and > 80 years old) patients (Additional File 6).

## Discussion

We sought to develop a general adult hospital prediction model that would identify, within the first 24 h of hospitalization, patients at the highest risk of requiring PAC services following discharge. We developed and internally validated a parsimonious prediction model that was well calibrated, and had high accuracy, and had an AUC of 0.875. Importantly, the prediction model exclusively utilized structured and readily available risk factors from the EHR, allowing for calculation of risk in the first 24 h. Such a model may allow hospital services to initiate earlier DP and better target case management, social work, and therapy services to those at highest risk of requiring PAC.

The current research builds upon previously published work that predicts PAC placement. Previous studies have focused on specific inpatient populations, including patients with coronary artery bypass graft surgery [9], lower limb fractures [10], acute myocardial infarction [16], older age [7, 8], or internal medicine patients [6, 7, 24]. Our study is unique in that it is a generalizable model that applies to all adult hospitalized patients and performs with equal or better predictive ability as compared to previously published models. For example, a model developed on older medical inpatients, utilizing an in person questionnaire that assessed activities of daily living (ADL) had an AUC of 0.81 [6]. Another recent model developed upon medical inpatients that utilized nurse intake ADL information has an AUC of 0.82 [7]. Our study confirms the importance of functional data to predict PAC discharge, and demonstrates the ability to apply it broadly across medical and surgical populations. We improve upon previous models by avoiding reliance on an additional functional assessment which would need to be conducted at admission. Our model not only uses age, but also uses markers of frailty such as the Braden Score to help generate predictions based on factors likely to influence need for post-acute care. The model continues to offer useful negative predictive information for older populations based on our analyses of model performance in these cohorts (Additional File 6).

By including the entire adult hospital population, this model could allow for a hospital to more holistically measure and guide resources which are often shared across services lines (e.g., case management, social work, physical therapy). In addition, it allows for the implementation of a single model into the informatics infrastructure, rather than unique models for each care area. The value of the model will be greatest in clinical areas with highest risk factor burden including increasing medication counts, fall risk, and advancing age. The one service area, as demonstrated in sensitivity analyses, for which this model would not provide additional guidance to DP is obstetrics and gynecology. These patients are, not surprisingly, at substantially reduced risk for PAC, as a large proportion of such admissions are for uncomplicated deliveries. This does not, however, diminish the validity with which it can be applied to the remaining medical and surgical populations.

Some may feel that prediction of PAC discharge is intuitive and does not require an automated score. However, the utility of an automated tool is to point busy health team members towards patients who would benefit most from early DP when the clinician may not have activated appropriate resources to arrange for timely transfer. Previously published models predict PAC discharge with the inclusion of data that can only be identified after many days in the hospital, or even after discharge. This may include risk factors such as length of stay, administrative variables (e.g., ICD-9, ICD-10 codes) that are often coded after hospital discharge [8, 10, 16]. Using data available within 24 h of admission allows for real-time calculation, and therefore, can be clinically applied in real-time. Without an automatic trigger, the timing of case management, social work, or physical therapy initiation of care may be delayed on account of referral behaviors, of admission timing, the location of the patient, or even the order of a patient in a standard database (e.g., alphabetically) [7]. Given the model’s accuracy, particularly with regard to its negative predictive value, it was embedded into our center’s EHR and offers a prediction of PAC discharge for every patient on admission.

The predictor selection is another area that our model advances prior research, particularly in using routine nursing functional assessments This is not surprising when considering many prior models have demonstrated the relative importance of functional impairment in predicting PAC discharge^10,12,28^. Many functional predictors, however, require in-person research measurements or manually abstracted patient responses. Our current model extends the application of clinical measures that are markers for mobility, fall risk, and polypharmacy. The Braden Risk Score, Morse Fall Risk Score, and pre-admission medication are routinely measured for the clinical care purposes unrelated to predicting PAC risk, however, each are independently predictive of PAC discharge. We specifically chose these variables as they are commonly measured early during the hospital stay and have the potential to be generalizable to other hospitals that routinely measure these. An illness severity index was not necessary for creating a high-performing model, and may have added unnecessary complexity if these are not routinely calculated for all admissions.

Among the limitations of this analysis are the fact that it is a retrospective study that examines a diverse population but only at a single center which contains its own local discharge practices. It is possible that missing and misclassification of final discharge destination for some patients may have biased the model, however, the direction of the bias is not known and is again thought to be small. Though the AUC was high, the PPV of the model is low, in a practical sense, utilizing the model to define which patients require intensive, early discharge planning may result in misallocation of resources to some patients who will not eventually be discharged to PAC. While the random undersampling approach addressed the problem of class imbalance, the deletion of cases from the majority class may result in losing information. Hospice is a discharge destination that also requires significant early discharge planning. However, our model groups this discharge outcome as non-PAC discharge. This is because discharge planning for SNF and discharge planning for hospice overlap, but require different evaluations to organize placement. Furthermore, our model does not account for a growing emphasis on PAC which can be delivered in a home-based setting, [25, 26] nor does it incorporate data from after the COVID-19 pandemic, which had widespread impacts around transitions of care. Finally, our model is parsimonious and does not include alternative variables that could predict discharge destination (e.g., social determinants of health).

## Conclusions and implications

We have developed a PAC discharge prediction model for an adult hospital population. The model is parsimonious, includes EHR-derived data, and is calculated from data within 24 h of admission. Despite the limited number of variables and calculation early in the hospital stay, it is remarkably accurate with excellent calibration. Further research could externally validate as well as understand the impact of model calculations on changing and improving DP. As the model is deployed in the hospital EHR system, it may assist in targeting DP to the highest need patients and may improve the patient and provider experience of the overall discharge process.

## Supplementary Information

**Additional file 1.** Inclusion and exclusion criteria were applied to the population admitted during one calendar year, with an undersampling approach used to select cases and holdout cases.

**Additional file 2.** A histogram plot showing the distribution of medication counts in the derivation cohort.

**Additional file 3.** The post-acute care prediction model.

**Additional file 4.** A post-hoc sensitivity analysis of the model’s performance in medical vs. surgical populations.

**Additional file 5.** A post hoc sensitivity analysis of the model’s performance in obstetric/gynecology vs. non-obstetric gynecology patients.

**Additional file 6.** Performance matrix of implementing the predictive model on patients whose age were ≤ 55 Years Old.

## Data Availability

Data used in the generation of this model is stored in the Perioperative Data Warehouse at VUMC. The deidentified datasets analyzed during the current study are available from the corresponding author on reasonable request and with a data use agreement.

## Abbreviations

DP: Discharge Planning
PAC: Post-Acute Care
HER: Electronic Health Record
SNF: Skilled Nursing Facility
TRIPOD: The Transparent Reporting of a Multivariable Prediction Model for Individual Prognosis or Diagnosis
VUMC: Vanderbilt University Medical Center
HDW: Perioperative Data Warehouse
ICU: Intensive Care Unit
ED: Emergency Department
SD: Standard Deviation
IQR: Interquartile Range
OR: Odds Ratio
CI: Confidence Interval
PPV: Positive Predictive Value
NPV: Negative Predictive Value
AUC: Area Under the Curve
ROC: Receiver Operating Characteristic
