# Supplementary material for: Using phenotypic data from the Electronic Health Record (EHR) to predict discharge
Source: BMC Geriatr. 2023 Jul 11;23:424. doi: 10.1186/s12877-023-04147-y (PMC10334536; doi:10.1186/s12877-023-04147-y)
Supplement: Supplementary file 1 — Additional file 1. Inclusion and exclusion criteria were applied to the population admitted during one calendar year, with an undersampling approach used to select cases and holdout cases. [file 12877_2023_4147_MOESM1_ESM.pdf]

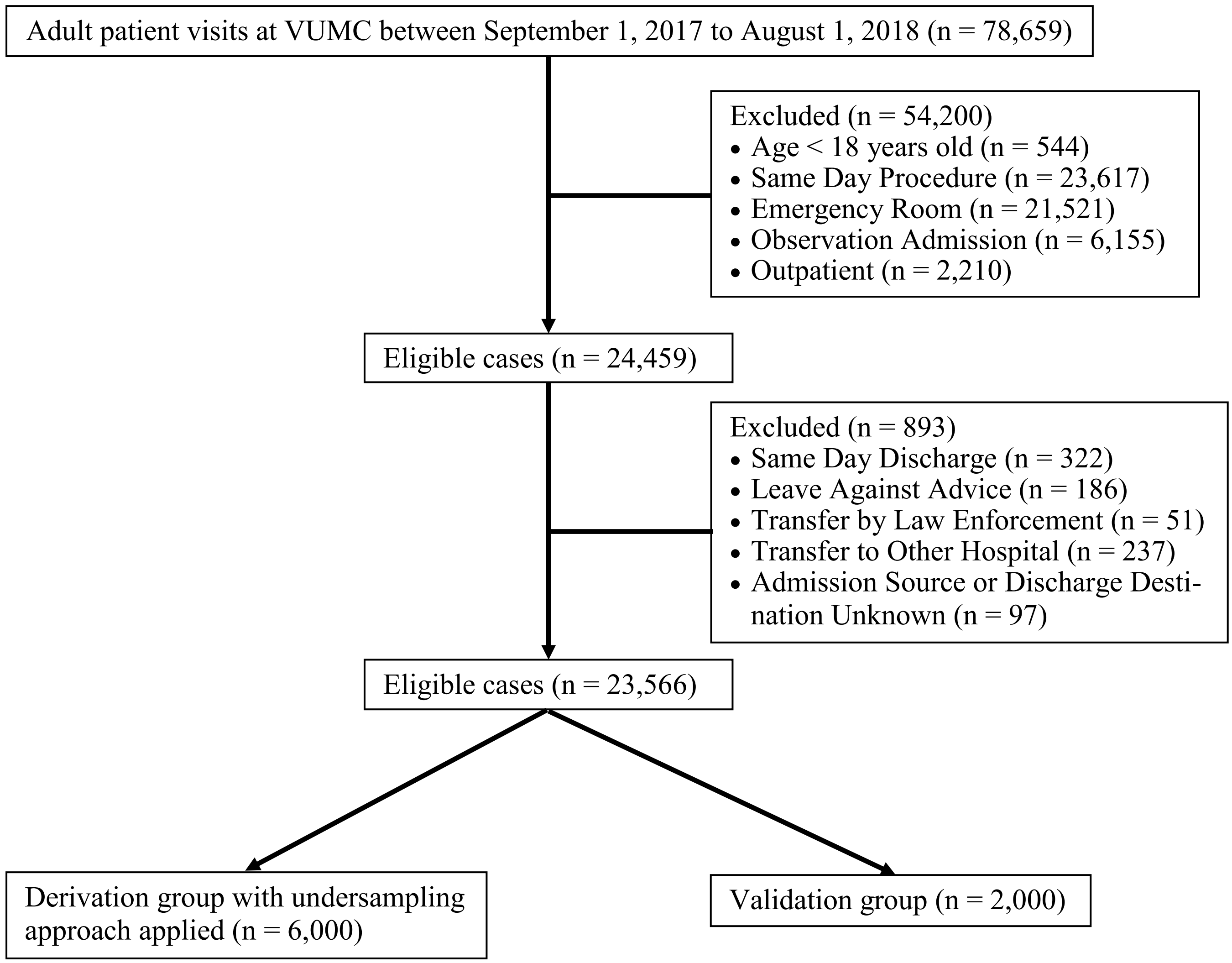

Supplementary Material 1. CONSORT flow diagram of inclusion/exclusion criteria. VUMC: Vanderbilt University Medical Center.

Additional File 1. Inclusion and exclusion criteria were applied to the population admitted during one calendar year, with an undersampling approach used to select cases and holdout cases.
