# Supplementary material for: Using phenotypic data from the Electronic Health Record (EHR) to predict discharge
Source: BMC Geriatr. 2023 Jul 11;23:424. doi: 10.1186/s12877-023-04147-y (PMC10334536; doi:10.1186/s12877-023-04147-y)
Supplement: Supplementary file 2 — Additional file 2. A histogram plot showing the distribution of medication counts in the derivation cohort. [file 12877_2023_4147_MOESM2_ESM.pdf]

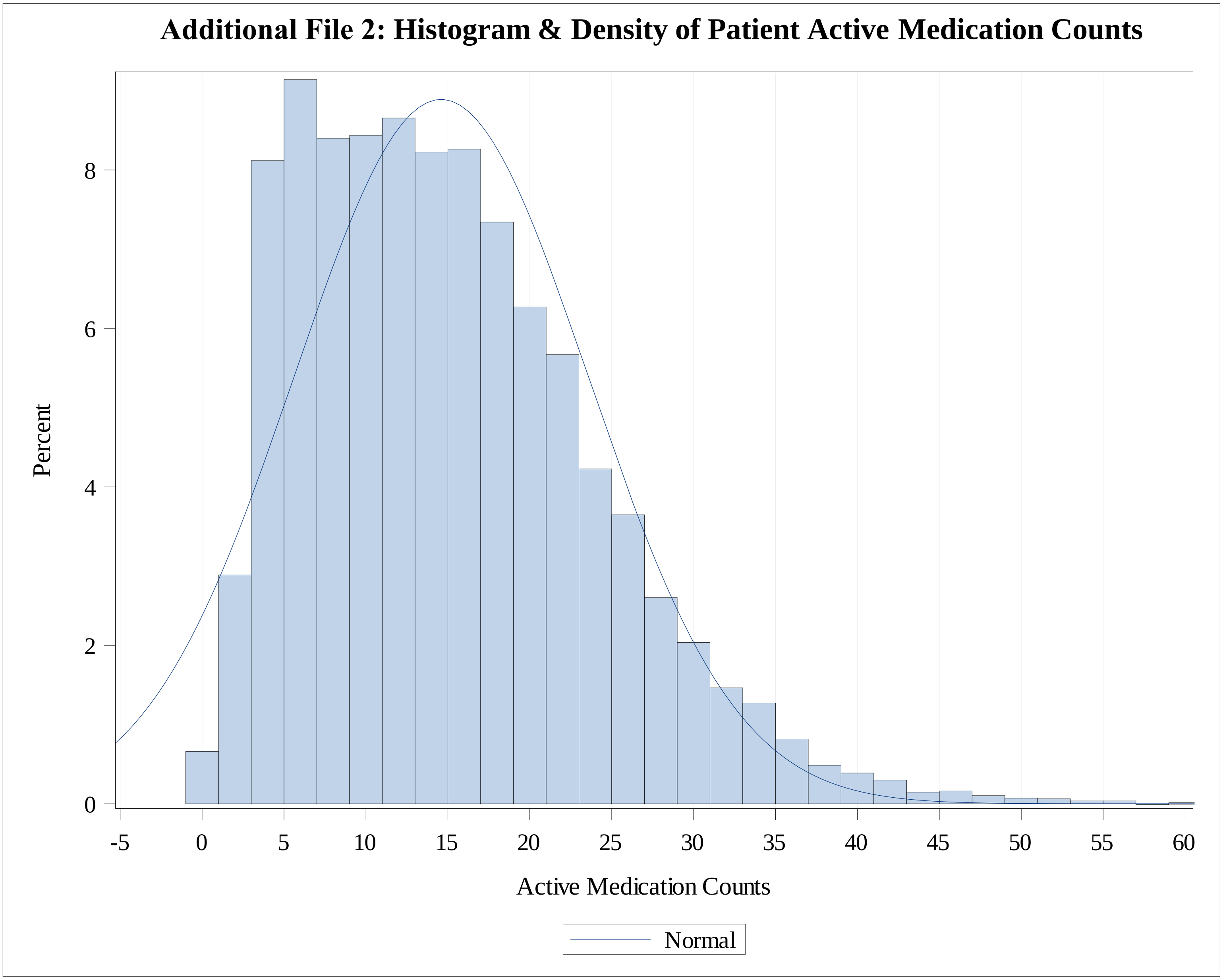

Additional File 2: A histogram plot showing the distribution of medication counts in the derivation cohort
