# Supplementary material for: Using phenotypic data from the Electronic Health Record (EHR) to predict discharge
Source: BMC Geriatr. 2023 Jul 11;23:424. doi: 10.1186/s12877-023-04147-y (PMC10334536; doi:10.1186/s12877-023-04147-y)
Supplement: Supplementary file 3 — Additional file 3. The post-acute care prediction model. [file 12877_2023_4147_MOESM3_ESM.docx]

**Additional File 3.** The post-acute care prediction model

The model can be summarized as follows:

$$logit=0.2380+0.0346\left( \mathrm{Age} \right)+0.0568\left( Active Medications \right)-0.24423\left( First Braden Score \right)+0.0244\left( First Fall Risk Score \right)-0.5398\left( Admitted from Home \right)-1.2597\left( Admitted from Clinic \right)+0.4239\left( Admitted from Emergency Department \right)+0.4097\left( Admitted to ICU \right)-1.5541(Gynecology Admission)$$
