# Supplementary material for: Using phenotypic data from the Electronic Health Record (EHR) to predict discharge
Source: BMC Geriatr. 2023 Jul 11;23:424. doi: 10.1186/s12877-023-04147-y (PMC10334536; doi:10.1186/s12877-023-04147-y)
Supplement: Supplementary file 4 — Additional file 4. A post-hoc sensitivity analysis of the model’s performance in medical vs. surgical populations. [file 12877_2023_4147_MOESM4_ESM.docx]

| **Additional File 4A. Performance Matrix of Implementing the Predictive Model on Surgical Cases.** | | | |
| --- | --- | --- | --- |
| Predicted Discharge Disposition | Observed Discharge Disposition | | |
| Frequency (N) | Post-acute Care | Home |  |
| Post-acute Care | 107 | 185 | Positive Predictive Value = 36.6% |
| Home | 22 | 696 | Negative Predictive Value = 96.9% |
| Total | Sensitivity = 83.0% | Specificity = 79.0% |  |

**Additional File 4.** A post-hoc sensitivity analysis of the model’s performance in medical vs. surgical populations

| **Additional File 4B. Performance Matrix of Implementing the Predictive Model on Non-Surgical Cases.** | | | |
| --- | --- | --- | --- |
| Predicted Discharge Disposition | Observed Discharge Disposition | | |
| Frequency (N) | Post-acute Care | Home |  |
| Post-acute Care | 115 | 158 | Positive Predictive Value = 42.1% |
| Home | 21 | 696 | Negative Predictive Value = 97.1% |
| Total | Sensitivity = 84.6% | Specificity = 81.5% |  |
