# Supplementary material for: Using phenotypic data from the Electronic Health Record (EHR) to predict discharge
Source: BMC Geriatr. 2023 Jul 11;23:424. doi: 10.1186/s12877-023-04147-y (PMC10334536; doi:10.1186/s12877-023-04147-y)
Supplement: Supplementary file 5 — Additional file 5. A post hoc sensitivity analysis of the model’s performance in obstetric/gynecology vs. non-obstetric gynecology patients. [file 12877_2023_4147_MOESM5_ESM.docx]

| **Additional File 5A. Performance Matrix of Implementing the Predictive Model on Obstetric Cases.** | | | |
| --- | --- | --- | --- |
| Predicted Discharge Disposition | Observed Discharge Disposition | | |
| Frequency (N) | Post-acute Care | Home |  |
| Post-acute Care | 0 | 0 | Positive Predictive Value = ∞ |
| Home | 1 | 249 | Negative Predictive Value = 99.6% |
| Total | Sensitivity = 0% | Specificity = 100% |  |

| **Additional File 5B. Performance Matrix of Implementing the Predictive Model on Non-Obstetric Cases.** | | | |
| --- | --- | --- | --- |
| Predicted Discharge Disposition | Observed Discharge Disposition | | |
| Frequency (N) | Post-acute Care | Home |  |
| Post-acute Care | 215 | 331 | Positive Predictive Value = 39.4% |
| Home | 49 | 1155 | Negative Predictive Value = 95.9% |
| Total | Sensitivity = 81.4% | Specificity = 77.7% |  |

**Additional File 5.** A post hoc sensitivity analysis of the model’s performance in obstetric/gynecology vs. non-obstetric gynecology patients.
