# Supplementary material for: Using phenotypic data from the Electronic Health Record (EHR) to predict discharge
Source: BMC Geriatr. 2023 Jul 11;23:424. doi: 10.1186/s12877-023-04147-y (PMC10334536; doi:10.1186/s12877-023-04147-y)
Supplement: Supplementary file 6 — Additional file 6. Performance matrix of implementing the predictive model on patients whose age were ≤ 55 Years Old. [file 12877_2023_4147_MOESM6_ESM.docx]

| **Supplementary Material. Performance Matrix of Implementing the Predictive Model on Patients whose Age were ≤ 55 Years Old.** | | | |
| --- | --- | --- | --- |
| Predicted Discharge Disposition | Observed Discharge Disposition | | |
| Frequency (N) | Post-acute Care | Home |  |
| Post-acute Care | 39 | 180 | Positive Predictive Value  17.8% |
| Home | 9 | 769 | Negative Predictive Value  98.8% |
|  | Sensitivity  81.3% | Specificity  81.0% |  |

| **Supplementary Material. Performance Matrix of Implementing the Predictive Model on Patients whose Age were Between 55 and 65 Years Old.** | | | |
| --- | --- | --- | --- |
| Predicted Discharge Disposition | Observed Discharge Disposition | | |
| Frequency (N) | Post-acute Care | Home |  |
| Post-acute Care | 48 | 64 | Positive Predictive Value  42.8% |
| Home | 9 | 287 | Negative Predictive Value  97.0% |
|  | Sensitivity  84.2% | Specificity  81.8% |  |

| **Supplementary Material. Performance Matrix of Implementing the Predictive Model on Patients whose Age were Between 65 and 80 Years Old.** | | | |
| --- | --- | --- | --- |
| Predicted Discharge Disposition | Observed Discharge Disposition | | |
| Frequency (N) | Post-acute Care | Home |  |
| Post-acute Care | 76 | 103 | Positive Predictive Value  42.5% |
| Home | 27 | 261 | Negative Predictive Value  90.6% |
|  | Sensitivity  73.8% | Specificity  71.7% |  |

| **Supplementary Material. Performance Matrix of Implementing the Predictive Model on Patients whose Age were > 80 Years Old.** | | | |
| --- | --- | --- | --- |
| Predicted Discharge Disposition | Observed Discharge Disposition | | |
| Frequency (N) | Post-acute Care | Home |  |
| Post-acute Care | 42 | 22 | Positive Predictive Value  65.6% |
| Home | 15 | 49 | Negative Predictive Value  76.6% |
|  | Sensitivity  73.7% | Specificity  69.0% |  |
